# Sudden Cause of Cardiac Death—Be Aware of Me: A Case Report and Short Review on Brugada Syndrome

**DOI:** 10.1155/2010/823490

**Published:** 2010-12-14

**Authors:** Jagadeesh K. Kalavakunta, Vishwaroop Bantu, Hemasri Tokala, Mihas Kodenchery

**Affiliations:** ^1^Kalamazoo Center for Medical Studies, Michigan State University, Kalamazoo, MI 49008, USA; ^2^Michigan State University, East Lansing, MI 48824, USA

## Abstract

*Introduction*. Brugada syndrome accounts for about 4% of sudden cardiac deaths (SCD). It is characterized by an ST-segment elevation in the right precordial electrocardiogram (EKG) leads. *Case Presentation*. We describe a 39-year-old healthy Caucasian man who was admitted to the intensive care unit after being cardioverted from ventricular fibrillation (VF) arrest. His past history was significant for an episode of syncope one month prior to this presentation for which he was admitted to an outlying hospital. EKG during that admission showed ST elevations in V1 and V2 leads, a pattern similar to Type 1 Brugada. A diagnosis of Brugada syndrome was missed and the patient had a cardiac arrest a month later. We discuss a short review of Brugada syndrome and emphasize the need to look for it in patients presenting with SCD and malignant arrhythmias. *Conclusion*. Physicians should always consider Brugada syndrome in the differential diagnosis of ST-segment elevation in anterior precordial leads of EKG and associated VT/VF. Although more than 17 years have passed since the first case was reported, increased awareness of this syndrome is needed to identify patients with EKG changes and treat them accordingly to prevent incidence of (SCD) and its deleterious complications.

## 1. Introduction

Brugada syndrome is a clinical entity first described in 1992 [1]. It is an autosomal dominant genetically predisposed disorder, characterized by an ST-segment elevation in the right precordial EKG leads and a high incidence of sudden cardiac death (SCD) in patients with structurally normal hearts. Almost 4% of patients presenting with SCD have Brugada syndrome, and therefore it should be looked for in patients with SCD. It is more common in males than females and typically first presents in the third decade of life, although it has been reported in children and the elderly too [2]. We present a case whose diagnosis is missed in the early presentation and developed SCD and its sequelae. 

## 2. Case Description

**Figure 1:**
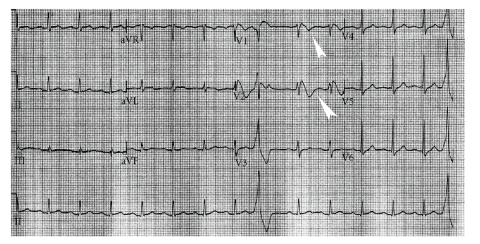
Electrocardiogram showing descendent ST-segment elevation with negative T waves (white arrow heads) in the right precordial leads V1 and V2.

A 39-year-old Caucasian man, with a past medical history of hypothyroidism, on synthroid, and no prior history of coronary artery disease, was in his usual state of health until he suddenly developed abnormal respiration and loud snoring while sleeping. His wife, who was awakened by the loud snort, found him unresponsive. Immediately she called emergency medical services (EMS) and initiated cardiopulmonary resuscitation. EMS arrived 10 minutes later and found the patient to be in ventricular fibrillation. He was successfully converted to sinus rhythm by multiple external defibrillations. He was intubated in the field and was then brought to the intensive care unit and initiated on a hypothermia protocol. After reviewing the records from his previous hospitalization, a significant episode of syncope occurring one month prior to this presentation was discovered. Electrocardiogram (EKG) at that admission showed sinus rhythm with incomplete right bundle branch block and persistent ST elevation in V1 and V2 leads, a pattern similar to type 1 Brugada syndrome (Figure 1). 

During this admission he was ruled out for myocardial infarction with serial cardiac biomarkers and was followed with further workup. His initial transthoracic echocardiography (TTE) was significant for ejection fraction (EF) of 25% with global hypokinesis. Subsequent transesophageal echocardiography (TEE) showed normal left ventricular function with ejection fraction of 65%. A cardiac catheterization revealed no significant coronary artery disease. During the entire admission the patient was in sinus rhythm and developed visual changes secondary to anoxic brain injury. An implantable cardioverter-defibrillator (ICD) placement was done as per current recommendations and the patient was subsequently discharged.

## 3. Discussion

Brugada syndrome has three types according to the EKG patterns. Type 1 has a “coved-” type ST elevation with at least 2 mm J-point elevation, a gradually descending ST-segment and a negative T wave in more than one right precordial leads (V1–V3). Type 2 has a “saddleback” pattern with at least 2 mm J-point elevation, and at least 1 mm ST elevation, with a positive or biphasic T wave. Type 2 pattern can occasionally be seen in healthy subjects. Type 3 has a saddle back or coved pattern with less than 2 mm J-point elevation, and less than 1 mm ST elevation, with a positive T wave. Type 3 pattern is not uncommon in healthy subjects. The type 2 and type 3 Brugada patterns are not specific enough to be considered diagnostic [2]. 

The pathophysiology of the Brugada Syndrome is still in debate between depolarization and repolarization hypothesis. According to Wilde et al. there is compelling evidence favoring repolarization hypothesis [3]. Dominant loss-of-function mutations in cardiac myocyte sodium channel encoding gene, named SCN5A [4], have been well documented in several cases. Other mutations in Glycerol-3-phosphate dehydrogenase-like peptide, L-type calcium channel (CACNA1c and its *β* subunit CACNB2b), and potassium outward current channel also have an association with Brugada syndrome [5]. New mutations are being added to the current literature on regular basis. Genetic testing for Brugada syndrome may confirm a diagnosis in patients suspected of having Brugada syndrome, as well as differentiate between relatives who are at risk for the disease and those who are not.

The Brugada pattern is a dynamic EKG finding, and it may not always appear on 12-lead EKG. A drug challenge test is used to establish the diagnosis; however, this test is not required if the type-1 Brugada pattern exists on the 12-lead EKG. Ajmaline, flecainide, pilsicainide, procainamide, disopyramide, and propafenone are the drugs utilized to unmask Brugada syndrome [2, 6]. It is definitively diagnosed when a type 1 ST-segment elevation is observed in >1 right precordial lead (V1 to V3) in the presence or absence of a sodium channel-blocking agent, and in conjunction with one of the following: documented ventricular fibrillation (VF), polymorphic ventricular tachycardia (VT), a family history of sudden cardiac death at <45 years old, coved-type EKGs in family members, syncope, or nocturnal agonal respiration [7].

The Brugada syndrome becomes unmasked in association with tricyclic antidepressant use, vagotonic agents, cocaine, and alcohol intoxication and in a febrile state [8, 9]. This is the reason why all the Brugada syndrome patients need to be educated about the aggressive treatment of fever and the medications to be avoided. ICD placement is the main stay of treatment as pharmacologic treatment has no mortality benefit. DEBUT study has shown that ICD treatment is superior to beta blockers [10]. New pharmacological agents are still under experimental studies. Symptomatic patients displaying the type 1 Brugada EKG (either spontaneously or after sodium channel blockade), who present with aborted sudden death, should receive an ICD without additional need for electrophysiologic study (EPS). 

Asymptomatic patients can be divided into 2 main categories: (1) those with a spontaneously occurring type 1 Brugada pattern and (2) those showing a type 1 Brugada pattern after a drug challenge. Role of EPS inducibility among asymptomatic patients is controversial. In spite of one third of asymptomatic patients having inducible ventricular arrhythmia, it does not seem to be equal to risk [11]. In the past, asymptomatic Brugada syndrome patients were associated with poor prognosis. Recent FINGER Brugada syndrome registry showed that in asymptomatic individuals the cardiac event rate per year was only 0.5%. Symptoms and spontaneous Type 1 Brugada syndrome are the strong predictors of future arrhythmic events. Gender, familial history of SCD, presence of an SCN5A mutation, and inducibility of ventricular arrhythmia were not predictive of arrhythmic events [12].

## 4. Conclusions

Physicians, especially emergency department and general physicians, should be aware of Brugada EKG patterns in the differential diagnosis of ST-segment elevation in anterior precordial leads of EKG. In a majority of the cases, and especially in young patients, consultation with a cardiologist or electrophysiologist is required. In this case, early diagnosis and prompt intervention might prevent SCD and subsequent sequelae.
